# Efficient Illumination for a Light-Addressable Potentiometric Sensor

**DOI:** 10.3390/s22124541

**Published:** 2022-06-16

**Authors:** Tatsuo Yoshinobu, Ko-ichiro Miyamoto

**Affiliations:** 1 Department of Biomedical Engineering, Tohoku University, Sendai 980-8579, Japan; 2 Department of Electronic Engineering, Tohoku University, Sendai 980-8579, Japan; koichiro.miyamoto.d2@tohoku.ac.jp

**Keywords:** light-addressable potentiometric sensor, LAPS, pH sensor, field-effect device, photocurrent, modulated illumination, lock-in detection, waveform, square wave, duty ratio

## Abstract

A light-addressable potentiometric sensor (LAPS) is a chemical sensor that is based on the field effect in an electrolyte–insulator–semiconductor structure. It requires modulated illumination for generating an AC photocurrent signal that responds to the activity of target ions on the sensor surface. Although high-power illumination generates a large signal, which is advantageous in terms of the signal-to-noise ratio, excess light power can also be harmful to the sample and the measurement. In this study, we tested different waveforms of modulated illuminations to find an efficient illumination for a LAPS that can enlarge the signal as much as possible for the same input light power. The results showed that a square wave with a low duty ratio was more efficient than a sine wave by a factor of about two.

## 1. Introduction

A light-addressable potentiometric sensor (LAPS) [1,2] is a chemical sensor that is based on a semiconductor, with a surface that can be flexibly modified with various sensing materials, such as ionophores, enzymes, aptamers, receptors, and cells, to render it a versatile platform for the electrochemical sensing and imaging [3,4] of both inorganic and organic chemical species. Its potential application range is wide, ranging from materials science to biology and medicine, and researchers have recently devoted substantial efforts to developing cell-based sensors for biomedical applications [5,6].

A LAPS has a field-effect structure [7,8] similar to that of an ion-sensitive field-effect transistor (ISFET) [9]. In both devices, the distribution of the charge carriers at the insulator–semiconductor interface varies by the field effect in response to the activity of target ions on the sensor surface. A variation in the channel conductance of an ISFET is detected in the form of a drain current, while a variation in the width of the depletion layer of a LAPS is detected in the form of a photocurrent.

To read out the change in the depletion layer, the LAPS sensor plate must be illuminated by a light beam with photon energy that is larger than the energy bandgap of the semiconductor. In most cases, the sensor plate is illuminated from the back surface to avoid the absorption or scattering of light by the sample on the front surface. Electron–hole pairs are generated by the absorption of light in the vicinity of the back surface, and they diffuse towards the insulator–semiconductor interface [10]. The electrons and holes are separated by the electric field inside the depletion layer, which functions as a current source [11]. Because the DC current is blocked by the insulator, the light beam is modulated to generate an AC photocurrent signal, and its amplitude correlates with the activity of target ions.

For a high-precision measurement, the signal should be as large as possible [12]. An increase in the input light power is a direct approach to obtaining a large signal from a LAPS, but excess light power is not only wasteful but also harmful to the measurement. As an extreme case, if the photon energy is so high as to cause ionization/deionization in the insulator or at the insulator–semiconductor interface, as in the case of a vacuum ultraviolet light, it can alter the properties of the field-effect structure, including the flat-band condition [13]. Even in the case of visible light, most of the light power is eventually converted into heat inside the semiconductor layer, which raises the temperature of the sensor plate. Both the charge-carrier properties of the semiconductor and the Nernst potential that is built up at the solution–insulator interface are responsive to temperature change, which may result in the drift in the slope sensitivity. In addition, a higher intensity of illumination not only increases the minority carriers that contribute to the photocurrent signal, but also raises the concentration of the majority carriers in the background. A device simulation of a LAPS revealed that this effect reduces the thickness of the depletion layer and lowers the spatial resolution of chemical imaging by a LAPS [14]. Finally, when a LAPS is applied to an in vivo measurement (for example, in the brain of an animal [15]), the injection of energy in any form into the body must be minimized as a safety measure, as well as to avoid its potential influence on the living organism.

In this study, we tested different waveforms of illuminations to find an efficient illumination for a LAPS to maximize the photocurrent signal that is generated by the same light power or, equivalently, to minimize the light power that generates the same photocurrent signal.

## 2. Materials and Methods

**Figure 1:**
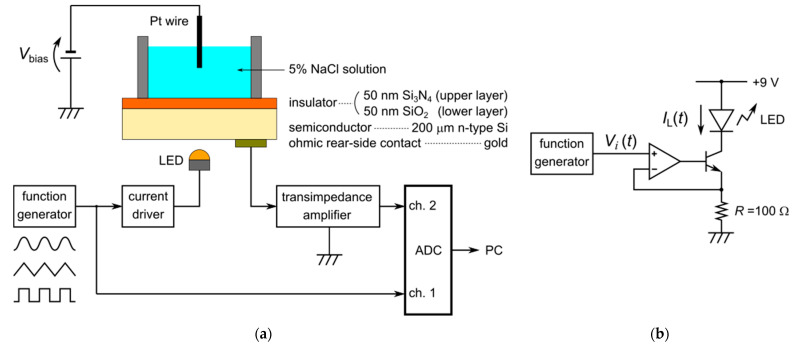
(**a**) Measurement setup for a LAPS. (**b**) LED current driver circuit.

The setup for the LAPS measurement that we used in this study is shown in Figure 1a. The LAPS sensor plate was composed of n-type Si, with a resistivity of 1–10 Ωcm, a size of 35 mm × 35 mm, and a thickness of 200 μm. We formed a 50 nm thick thermal oxide and deposited a 50 nm thick Si_3_N_4_, in this order, onto the front surface, and evaporated an Ohmic rear-side contact near the edge of the back surface.

Figure 1b shows the LED current driver circuit we used in this study. The input voltage ($V_{i}\left(t\right)$) controlled the LED current ($I_{L}\left(t\right)$), which is given by:

(1) $$I_{L}\left(t\right)=\frac{\beta }{1+\beta }\cdot \frac{V_{i}\left(t\right)}{R}\approx \frac{V_{i}\left(t\right)}{R},$$

where β (≫1) is the common-emitter current gain of the bipolar junction transistor. We can calculate the light power ($P_{L}\left(t\right)$) emitted by the LED as a product of the photon energy and the number of photons emitted in a unit of time:

(2) $$P_{L}\left(t\right)=\frac{hc}{\lambda }\cdot \eta \cdot \frac{I_{L}\left(t\right)}{q},$$

where h is Planck’s constant, c is the speed of light in a vacuum, λ is the wavelength of the light, η is the quantum yield of the LED, and q is the elementary charge. The light power is therefore proportional to the LED current ($I_{L}\left(t\right)$). In this study, we placed a 5 mm round-shaped amber LED (C503B-AAN-CY0B0251, Cree LED, Durham, NC, USA) with λ=591 nm in proximity to the back surface of the sensor plate, and we supplied the input voltage ($V_{i}\left(t\right)$) by a digital function generator (DF1906, NF Corporation, Yokohama, Japan), which generated various shapes of periodic functions with a specified frequency.

The transimpedance amplifier virtually grounded the sensor plate, and we applied a fixed bias voltage of −1.5 V across the field-effect structure, at which the vicinity of the insulator–semiconductor interface was in the inversion state. To minimize the influence of the frequency characteristics of the measurement circuit, we directly applied the bias voltage to a Pt wire dipped in 5% NaCl solution on the sensor surface by a DC voltage source instead of using an electrochemical potentiostat.

A wideband transimpedance amplifier (SA-604F2, NF corporation) amplified and converted the photocurrent signal into voltage, with a gain of 10^7^ V/A, and we set the cut-off frequency of the built-in low-pass filter at 30 kHz, which was sufficiently higher than the modulation frequencies used in this study. We digitally sampled the amplified signal, together with the input voltage ($V_{i}\left(t\right)$), at a sampling frequency of $f_{s}=100\;\mathrm{kHz}$, by a 16-bit analog-to-digital converter of the multifunction I/O device (USB-6341, National Instruments), and we recorded it with a PC using a program written with LabVIEW (National Instruments, Austin, TX, USA).

The photocurrent signal ($I_{\mathrm{sig}}\left(t\right)$) is essentially a periodic function with the same period (T) as that of the $I_{L}\left(t\right)$. To determine the amplitude of the $I_{\mathrm{sig}}\left(t\right)$, we used the principle of dual-phase lock-in detection, which extracts only the component that corresponds to the reference frequency. Lock-in detection is not only advantageous for the reduction in noise, but it is also indispensable in cases where more than one light beam modulated at different frequencies is employed to simultaneously address a plurality of locations on the sensor plate for high-speed measurement [16,17].

When we consider the Fourier series expansion of $I_{\mathrm{sig}}\left(t\right)$:

(3) $$I_{\mathrm{sig}}\left(t\right)=\frac{a_{0}}{2}+\overset{\infty }{\underset{n=1}{\sum }}\left(a_{n}\cos\frac{2\pi nt}{T}+b_{n}\sin\frac{2\pi nt}{T}\right),$$

(4) $$a_{n}=\frac{2}{T}\int_{0}^{T}I_{\mathrm{sig}}\left(t\right)\cos\frac{2\pi nt}{T}dt,$$

(5) $$b_{n}=\frac{2}{T}\int_{0}^{T}I_{\mathrm{sig}}\left(t\right)\sin\frac{2\pi nt}{T}dt,$$

the amplitude of the fundamental frequency of $I_{\mathrm{sig}}\left(t\right)$ (hereafter called $A_{\mathrm{sig}}$) is given by:

(6) $$A_{\mathrm{sig}}=\sqrt{a_{1}{}^{2}+b_{1}{}^{2}}.$$

Our goal, therefore, was to maximize the value of $A_{\mathrm{sig}}/\bar{I_{L}}$. Here, $\bar{I_{L}}$ is the average LED current, which is proportional to the average input light power.

For the calculation of $A_{\mathrm{sig}}$ from the experimentally obtained photocurrent signal, we always used 200 cycles of digitally sampled data ($I_{\mathrm{sig},\;1},\;I_{\mathrm{sig},\;2},\cdots I_{\mathrm{sig},\;N}$), where the number of samples was $N=200f_{s}T$. We then numerically calculated the values of $a_{1}$ and $b_{1}$ as:

(7) $$a_{1}=\frac{2}{N}\overset{N}{\underset{k=1}{\sum }}I_{\mathrm{sig},k}\;\cos\frac{2\pi k}{f_{s}T},$$

(8) $$b_{1}=\frac{2}{N}\overset{N}{\underset{k=1}{\sum }}I_{\mathrm{sig},k}\;\sin\frac{2\pi k}{f_{s}T},$$

from which we obtained $A_{\mathrm{sig}}$ by Equation (6).

## 3. Results and Discussion

**Figure 2:**
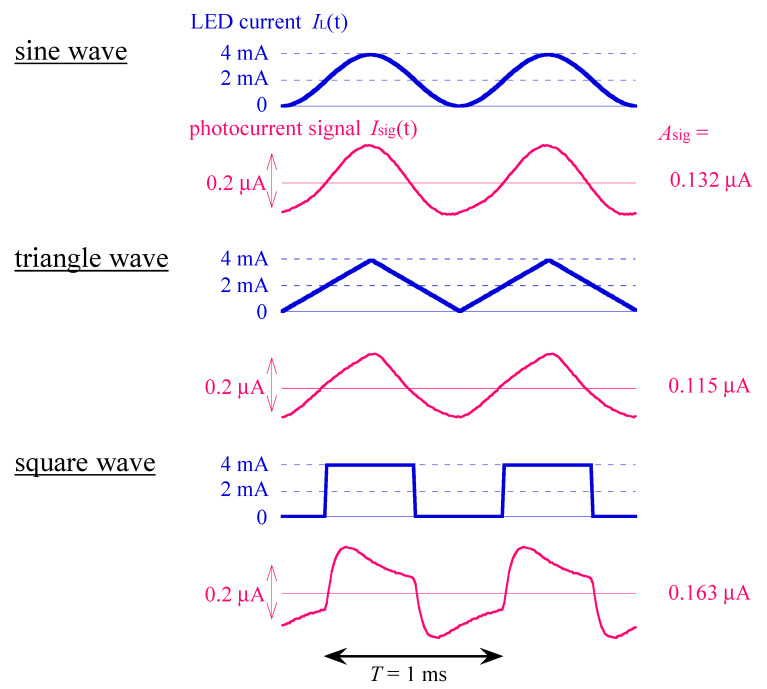
Waveforms of photocurrent signal ($I_{\mathrm{sig}}\left(t\right)$) (plotted in red) in response to different waveforms (sine, triangle, and square) of $I_{L}\left(t\right)$ (plotted in blue) with the same frequency (1 kHz) and the same average LED current (2 mA). The values of the amplitude of fundamental frequency ($A_{\mathrm{sig}}$ ) are also shown. All the waveforms shown in this figure are averages from over 100 cycles recorded.

First, we investigated the effect of the shape of $I_{L}\left(t\right)$ on the value of $A_{\mathrm{sig}}$. We tested three different waveforms, namely, a sine wave, a triangle wave, and a square wave, which are plotted in blue in Figure 2. All three waveforms had the same fundamental frequency (1 kHz) and the same average LED current ($\bar{I_{L}}$ of 2 mA). The maximum and the minimum current values were 4 and 0 mA, respectively. The resultant photocurrent signals ($I_{\mathrm{sig}}\left(t\right)$) for each waveform are plotted in red. Note that the photocurrent signal ($I_{\mathrm{sig}}\left(t\right)$) has no DC component because the DC current is blocked by the insulator layer.

The magnitude of $I_{\mathrm{sig}}\left(t\right)$ was approximately four orders smaller than that of $I_{L}\left(t\right)$. This ratio is mostly determined by the decay factor (exp(−d/L)), where d is the thickness of the semiconductor layer, and L is the diffusion length of the minority carriers (holes in the present case) [10,11]. The small value of this factor suggested that most of the photo carriers generated at the back surface of the sensor plate were lost by recombination in the course of diffusion across the sensor plate. The values of $A_{\mathrm{sig}}$ were 0.132, 0.115, and 0.163 μA for the sine, triangle, and square waves, respectively. The square wave resulted in the largest value of $A_{\mathrm{sig}}$ among these three waveforms, for the same value of $\bar{I_{L}}$.

This result can be understood by considering the amplitude of the fundamental frequency of the $I_{L}\left(t\right)$ (hereafter called $A_{L}$). We can calculate the values of the $A_{L}$ for the sine, triangle, and square waves as follows:

(9) $$\mathrm{sine}\;\mathrm{wave}\;A_{L}=\frac{2}{T}\cdot \int_{0}^{T}\;\bar{I_{L}}\cdot \sin\frac{2\pi t}{T}\cdot \sin\frac{2\pi t}{T}dt=\bar{I_{L}},\;\;\;\;\;\;\;\;\;\;\;\;\;\;\;\;\;\;\;\;\;\;\;\;\;\;\;\;\;\;\;\;\;\;\;\;\;\;$$

(10) $$\mathrm{triangle}\;\mathrm{wave}\;A_{L}=\frac{2}{T}\cdot 4\int_{0}^{\frac{T}{4}}\;\frac{\bar{I_{L}}\cdot t}{T/4}\cdot \sin\frac{2\pi t}{T}dt=\frac{8}{\pi^{2}}\cdot \bar{I_{L}}\approx 0.811\cdot \bar{I_{L}},\;\;\;\;\;\;\;\;\;\;\;\;\;\;\;$$

(11) $$\mathrm{square}\;\mathrm{wave}\;A_{L}=\frac{2}{T}\cdot \int_{0}^{\frac{T}{2}}\;2\cdot \bar{I_{L}}\cdot \sin\frac{2\pi t}{T}dt=\frac{4}{\pi }\cdot \bar{I_{L}}\approx 1.27\cdot \bar{I_{L}}.\;\;\;\;\;\;\;\;\;\;\;\;\;\;\;\;\;\;\;\;\;\;\;$$

The ratios among them were in good agreement with the ratios among the experimentally obtained values of $A_{\mathrm{sig}}$ (0.115/0.132=0.871 and 0.163/0.132=1.23), which suggested that $A_{\mathrm{sig}}$ was primarily determined by $A_{L}$ despite the nonlinear distortion of waveforms.

From a practical point of view, a square wave of $I_{L}\left(t\right)$ is much easier to generate than a sine or a triangle wave, as it requires only one bit of output from a digital counter circuit to periodically switch the LED current on and off. This advantage increases in the case where a large number of light beams must be simultaneously controlled. An array of digital counters can be implemented, for example, in a single chip of a field-programmable gate array to output square waves [18].

**Figure 3:**
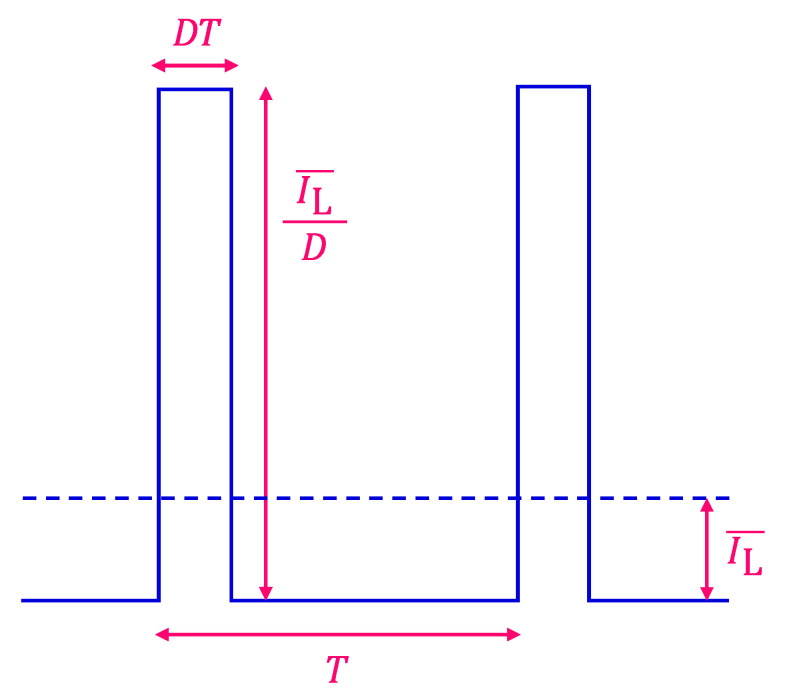
Square wave of $I_{L}\left(t\right)$ with period (T), duty ratio (D), and an average LED current ($\bar{I_{L}}$). Peak pulse height is $\bar{I_{L}}/D$.

We could further increase the value of $A_{L}$ by changing the duty ratio of a square wave. Figure 3 shows a square wave with a duty ratio (D) and an average LED current ($\bar{I_{L}}$):

The value of $A_{L}$, in this case, is calculated as follows:

(12) $$\mathrm{square}\;\mathrm{wave}\;\left(\mathrm{duty}\;\mathrm{ratio}\;\left(D\right)\right)\;\;\;\;\;A_{L}=\frac{2}{T}\cdot 2\int_{0}^{\frac{DT}{2}}\;\frac{\bar{I_{L}}}{D}\cdot \cos\frac{2\pi t}{T}dt=\frac{2\sin D\pi }{D\pi }\cdot \bar{I_{L}}.\;\;\;\;\;\;\;\;\;\;\;\;\;\;\;\;\;\;$$

The factor 2sinDπ/Dπ coincides with the value 4/π in Equation (11) at D=0.5, and asymptotically approaches 2 in the limit of D→0, which is the case of a periodic delta function. The peak height of the pulse becomes larger as D becomes smaller, but it is limited in practice by the absolute maximum current of the LED. In this study, we reduced the value of D to 0.20, while always maintaining the value of $\bar{I_{L}}$ constant at 2 mA.

**Figure 4:**
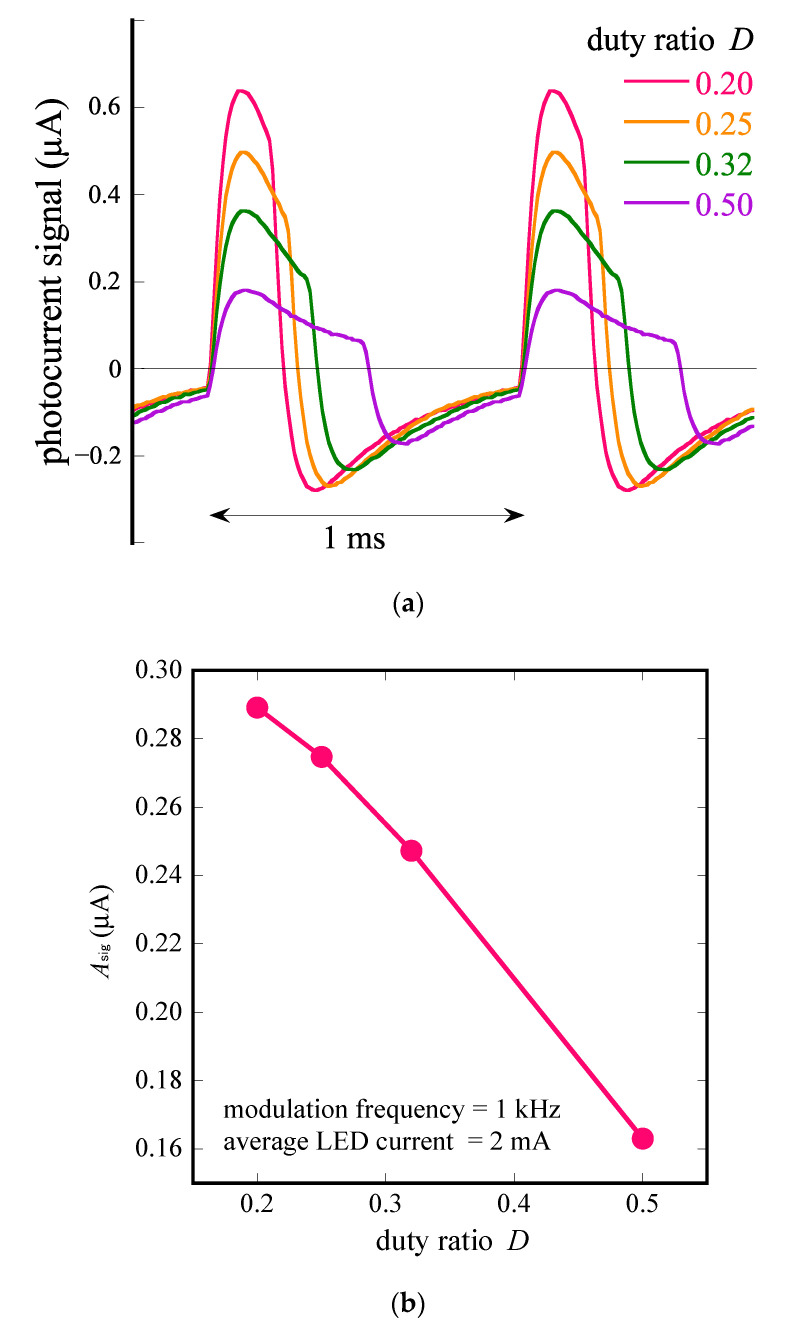
(**a**) Waveforms of $I_{\mathrm{sig}}\left(t\right)$ for different duty ratios (0.20, 0.25, 0.32, and 0.50) of square waves of $I_{L}\left(t\right)$ with the same frequency (1 kHz) and the same average LED current (2 mA). All waveforms shown were averaged over 100 cycles. (**b**) Amplitude of fundamental frequency of photocurrent signal ($A_{\mathrm{sig}}$), plotted as a function of duty ratio (D ).

Figure 4a shows the experimentally obtained photocurrent signals ($I_{\mathrm{sig}}\left(t\right)$) for D= 0.20, 0.25, 0.32, and 0.50. As D becomes smaller, the pulses become narrower and taller.

In Figure 4b, we plot the amplitude of the fundamental frequency of the photocurrent signal ($A_{\mathrm{sig}}$) as a function of the duty ratio (D). As we expected, the amplitude ($A_{\mathrm{sig}}$) increased as D reduced. The value of $A_{\mathrm{sig}}$ at D= 0.20 was 0.289 μA, which was slightly larger than twice the value for a sine wave (0.132 μA). A further reduction in D would result in even taller pulses, which, however, does not contribute to a substantial increase in $A_{\mathrm{sig}}$. However, a higher peak value demands more allowance for both the output current of the LED current driver and the input range of the trans-impedance amplifier. Therefore, from a practical point of view, a duty ratio of 0.20 is an appropriate compromise.

**Figure 5:**
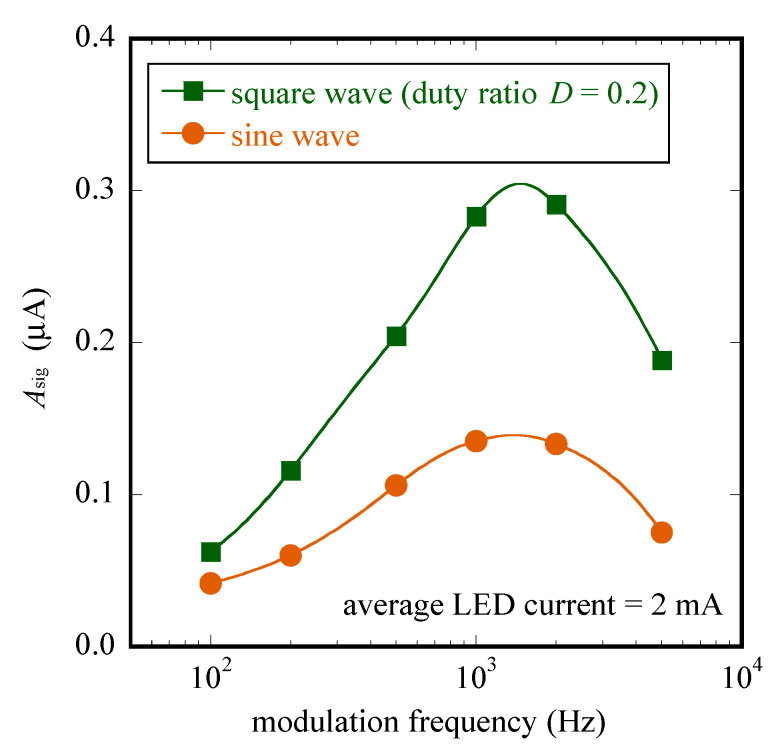
Comparison of values of the amplitude of fundamental frequency ($A_{\mathrm{sig}}$) obtained with a sine and a square wave with a low duty ratio (D=0.2) in a frequency range from 100 to 5000 Hz.

Finally, Figure 5 compares the values of $A_{\mathrm{sig}}$ we obtained with a sine wave and a square wave (D=0.2) of illumination in a typical frequency range of a LAPS, 100 to 5000 Hz. The overall shape of the frequency dependence is typical for a conventional LAPS sensor plate; the photocurrent had a peak in the kHz region and decayed at both lower and higher frequencies [10,11,19]. Except for the lowest frequency, the photocurrent generated by a square wave (D=0.2) of illumination was always larger than a sine wave by a factor of about two. This result showed that the correct choice of the modulation frequency, as well as the waveform, results in a much higher photocurrent signal, which is advantageous for high-precision measurement with a LAPS sensor plate.

## 4. Conclusions

In this study, we tested different waveforms of illumination in the search for an efficient illumination for a LAPS that maximizes the photocurrent signal for the same input light power. We found that a square wave with a low duty ratio could generate a larger photocurrent signal than a sine wave by a factor of about two throughout the typical frequency range of a LAPS sensor plate. The correct choice of the modulation frequency, as well as the waveform, is important to maximize the efficiency of the signal generation and to obtain a higher signal-to-noise ratio in LAPS measurement.
